# In-Situ Hydrothermal Synthesis of Bi–Bi_2_O_2_CO_3_ Heterojunction Photocatalyst with Enhanced Visible Light Photocatalytic Activity

**DOI:** 10.1007/s40820-016-0118-0

**Published:** 2016-12-02

**Authors:** Prasenjit Kar, Tuhin Kumar Maji, Ramesh Nandi, Peter Lemmens, Samir Kumar Pal

**Affiliations:** 1 Department of Chemical Biological and Macromolecular Sciences, S. N. Bose National Centre for Basic Sciences, Block JD, Sector III, SaltLake, Kolkata, 700106 India; 2 Institute for Condensed Matter Physics, TU Braunschweig, Mendelssohnstraße 3, 38106 Brunswick, Germany; 3 Laboratory for Emerging Nanometrology, TU Braunschweig, Brunswick, Germany

**Keywords:** Bi nanoparticles, Bi–Bi_2_O_2_CO_3_ nanosheets, Heterojunction, Hydrothermal method, Charge separation, Visible light photocatalytic activity

## Abstract

Bismuth containing nanomaterials recently received increasing attention with respect to environmental applications because of their low cost, high stability and nontoxicity. In this work, Bi–Bi_2_O_2_CO_3_ heterojunctions were fabricated by in-situ decoration of Bi nanoparticles on Bi_2_O_2_CO_3_ nanosheets *via* a simple hydrothermal synthesis approach. X-ray diffraction (XRD), scanning electron microscopy (SEM), transmission electron microscopy (TEM) and high-resolution TEM (HRTEM) were used to confirm the morphology of the nanosheet-like heterostructure of the Bi–Bi_2_O_2_CO_3_ composite. Detailed ultrafast electronic spectroscopy reveals that the in-situ decoration of Bi nanoparticles on Bi_2_O_2_CO_3_ nanosheets exhibit a dramatically enhanced electron-hole pair separation rate, which results in an extraordinarily high photocatalytic activity for the degradation of a model organic dye, methylene blue (MB) under visible light illumination. Cycling experiments revealed a good photochemical stability of the Bi–Bi_2_O_2_CO_3_ heterojunction under repeated irradiation. Photocurrent measurements further indicated that the heterojunction incredibly enhanced the charge generation and suppressed the charge recombination of photogenerated electron-hole pairs.

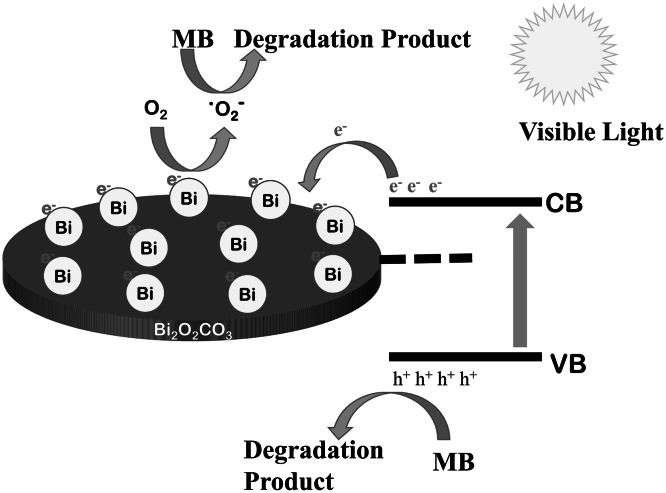

## Highlights

- A facile low cost hydrothermal technique was employed to synthesize of Bi-Bi_2_O_2_CO_3_, and Bi nanoparticles was decorated in-situ on Bi_2_O_2_CO_3_.
- The heterostructure exhibits enhanced electron-hole separation and improves visible-light photocatalytic activity effectively.

## Introduction

Photocatalysis technology has attracted enormous interest because of its potential to soften and release the global energy crisis and environmental pollution [1–6]. Although various types of semiconductor photocatalyst have been developed, their applications are impeded by a high recombination rate of electron–hole pairs and low efficiency of solar light absorption in the photocatalysis [7, 8]. A tremendous effort has been made to optimize the electronic band structure allowing an efficient electron–hole separation, which has been acknowledged to be a key factor in enhancing solar energy conversion [9–14]. The development of heterojunction systems has also been understood since it is beneficial for electron transfer to improve electron–hole pair separation, and therefore resulting in an excellent photocatalytic activity under solar light illumination [15, 16].

Recently, economic and abundant bismuth-containing semiconductors have been attracted large attention for diverse applications, especially in the area of energy conversion and environmental treatment [17, 18, 19, 20, 21]. The bismuth subcarbonate (Bi_2_O_2_CO_3_) is one of the most interesting semiconductor with a large bang gap of 3.3 eV. It belongs to the layered Aurivillius-related oxide family, consisting of Bi_2_O_2_^2+^ layers sandwiched between two slabs of CO_3_^2−^ layers [22]. However, the use of Bi_2_O_2_CO_3_ in light harvesting applications is very limited because it can absorb only UV light. To overcome this drawback, several 3D hierarchical Bi_2_O_2_CO_3_ architectures composed of nanosheets, nanoplates and microspheres have been developed [23, 24]. The coupling of Bi_2_O_2_CO_3_ with other materials to construct heterojunctions has also been shown as an advantages approach to improve the visible light responsive activity and to facilitate the separation of photogenerated electron–hole pairs. Different low band gap semiconductors and polymers have been used to improve the photocatalytic activity of Bi_2_O_2_CO_3_. Liu et al. constructed hierarchical graphene–Bi_2_O_2_CO_3_ composites which exhibit a significantly enhanced visible light photocatalytic performance [25]. Good visible light photocatalytic activity toward the degradation of Rhodamine B was reported by Zhang et al. for *n*–*n* heterostructured Bi_2_O_2_CO_3_/Bi_2_WO_6_ [26]. Zhou’s group reported that PANI decorated Bi_2_O_2_CO_3_ nanosheets exhibited a four to half times better photocatalytic activity for degradation of Rhodamine B in comparison to Bi_2_O_2_CO_3_ nanosheets under visible light illumination [27]. Recently, p–n heterojunction Ag_2_O/Bi_2_O_2_CO_3_ photocatalysts was shown to manifest an excellent visible light activity for degradation of MB and methyl orange [28].

Recently surface plasmon resonance (SPR) of noble metal nanoparticles (Ag or Au) was reported for improving the activity of semiconductor photocatalysts efficiently [29, 30]. In comparison with the high cost of noble-metals, Bi nanoparticles are inexpensive and show comparable SPR [31]. Recently, two reports on Bi nanoparticles demonstrate that they are useful for catalysis and sensing applications [32, 33]. Dong et al. showed that plasmonic Bi nanoparticles can be used for NO removal [34]. Several Bi nanoparticles based nanocomposites like Bi/BiOCl, Bi/Bi_2_O_3,_ and Bi/BiOI exhibit enhanced photocatalytic activity comparing to their counterpart [35–37]. Recently, Bi nanoparticles based heterojunctions with semiconductor have been an intense research area due to their enhanced charge separation and improved photocatalytic efficacies [38–40]. However, Bi nanoparticles decorated Bi_2_O_2_CO_3_ nanosheets have not been considered up to date.

In the present study, we developed an in situ decoration of Bi nanoparticles on Bi_2_O_2_CO_3_ nanosheets via a one-pot hydrothermal method. From time-resolved fluorescence spectroscopy, we observed that an ultrafast electron transfer process in the Bi–Bi_2_O_2_CO_3_ heterojunction reveals an excited state electron transfer from Bi_2_O_2_CO_3_ to Bi. The novel Bi-decorated Bi_2_O_2_CO_3_ nanosheets exhibited a dramatically enhanced photocatalytic activity towards MB degradation comparing to pure Bi_2_O_2_CO_3_ nanosheets because of the SPR effect of Bi nanoparticles and an efficient separation of electron–hole pairs in the Bi–Bi_2_O_2_CO_3_ heterojunction. We also observed that Bi–Bi_2_O_2_CO_3_ exhibited a good recyclability with respect to degradation of MB, which is significant for real world applications.

## Experimental Section

### Reagents

Bismuth nitrate pentahydrate [Bi(NO_3_)_3_·5H_2_O], cityl trimethyl ammonium bromide (CTAB), sodium carbonate (Na_2_CO_3_), and methylene blue (MB) were purchased from Sigma Aldrich. All other chemicals employed were of analytical grade and used without further purification.

### Synthesis of Bi–Bi_2_O_2_CO_3_ Heterojunction

In a typical synthesis of Bi–Bi_2_O_2_CO_3_, 0.2 millimol Bi(NO_3_)_3_·5H_2_O was first dissolved in 20 mL 1 M HNO_3_ (denoted as solution A). Meanwhile, 1.6 millimol Na_2_CO_3_ and 50 mg CTAB were dissolved in 20 mL ethanol–water mixture (denoted as solution B). Then, solution B was added into solution A under stirring for 30 min at 30 °C. The resulting mixture was transferred into a 20 mL Teflon-lined stainless-steel autoclave and was placed into an oven to react at 180 °C for 6 h. The system was then cooled to ambient temperature naturally. The final product was collected and washed with distilled water and absolute alcohol at least five times. As-prepared samples were dried at 60 °C for 6 h. The reductive nature of EtOH and CTAB allowed an in situ formation of Bi nanoparticles on the Bi_2_O_2_CO_3_ nanosheet. As a result, a heterojunction structure consisting of Bi_2_O_2_CO_3_ sheets and metallic Bi nanoparticles has been produced. For the synthesis of Bi_2_O_2_CO_3_ nanosheets we follow a preparation as reported by Zhou et al. [27].

### Characterization Methods

Field emission scanning electron microscopy (FESEM, QUANTA FEG 250) was used to investigate the surface morphology of the samples and samples were performed by applying a diluted drop of samples on a silicon wafer. Transmission electron microscopy (TEM) grids were prepared by applying a diluted drop of the samples to carbon-coated copper grids. The particle sizes were determined from micrographs recorded at a magnification of 100,000X using an FEI (Technai S-Twin, operating at 200 kV) instrument. X-ray diffraction (XRD) patterns of the samples were recorded by employing a scanning rate of 0.02° S^−1^ in the 2*θ* range from 20° to 80° using a PANalytical XPERTPRO diffractometer equipped with Cu *K*_α_ radiation (at 40 mA and 40 kV). For optical experiments, the steady-state absorption and emission were carried out with a Shimadzu UV-2600 spectrophotometer and a Jobin–Yvon Fluoromax-3 fluorimeter, respectively. Picosecond-resolved spectroscopic studies were carried out using a commercial time correlated single photon counting (TCSPC) setup from Edinburgh Instruments (instrument response function, IRF = 80 ps, excitation at 375 nm). The details of experimental setting up and methodology were described in our earlier reports [41, 42].

### Photocatalytic Performance Measurements

The photocatalysis activity of the samples were evaluated in terms of photodegradation of MB which was taken as a model pollutant in water. The photodegradation reaction of MB (initial concentration *C*_0_ = 0.5 × 10^−5^ M) was carried out in a 10 mm optical path quartz cell reactor containing 2 mL of a model MB solution with a concentration of 0.5 g L^−1^ of the photocatalyst in deionized water (DI). The suspension was irradiated with a mercury lamp, *λ* ≥ 400 nm (under visible light) and absorbance data were collected continuously by using a reported setting [4]. The percentage degradation (%DE) of MB was determined by Eq. 1:

1 $$\% \;{\text{DE}}\; = \frac{{I_{0} - I}}{{I_{0} }}\; \times \;100$$

where *I*_0_ is the initial absorption intensity of MB at *λ*_max_ = 660 nm and *I* is the absorption intensity after irradiation.

### Photocurrent Measurements

Photocurrent measurements were done in a dye-sensitized solar cell (DSSC) setup [43]. To prepare the working and counter electrodes for the photocurrent responses, FTO glasses were ultrasonically cleaned in soap-suds, deionized water, and acetone, respectively. For preparation of the counter electrode, platinum (Pt) was deposited on the FTO substrates by thermal decomposition of 10 mM platinum chloride (in isopropanol) at 385 °C for 30 min. Bi_2_O_2_CO_3_ and Bi–Bi_2_O_2_CO_3_ were used as the photoelectrode. The two electrodes were placed on top of each other with a single layer of 60 μm thick Surlyn (Solaronix) as a spacer between the two electrodes. A liquid electrolyte composed of 0.5 M lithium iodide (LiI), 0.05 M iodine (I_2_) and 0.5 M 4-*tert*-butylpyridine (TBP) in acetonitrile was used as the hole conductor and filled in the inter electrode space using capillary force through two small holes (diameter = 1 mm) predrilled on the counter electrode. Finally, the two holes were sealed by using another piece of Surlyn to prevent a leakage of the electrolyte from the cell. In all our experiments, the active area of the DSSCs was fixed to 1 cm^2^.

## Results and Discussion

**Fig. 1:**
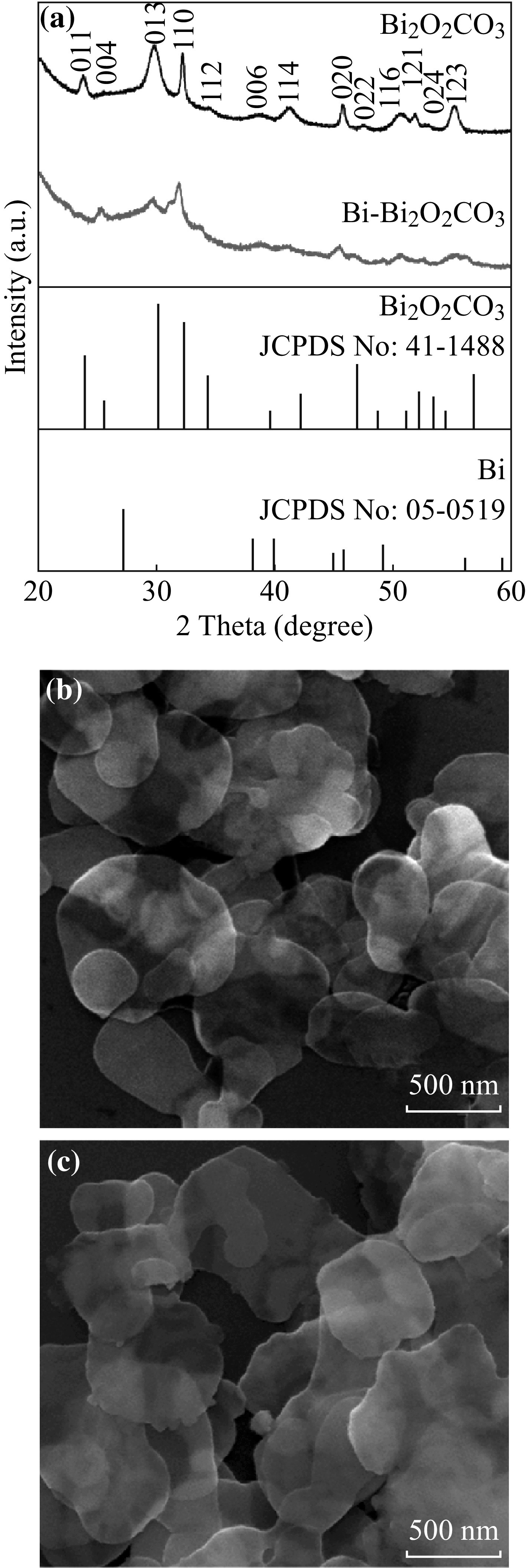
**a** XRD patterns of Bi_2_O_2_CO_3_ and Bi–Bi_2_O_2_CO_3_. SEM images of **b** Bi_2_O_2_CO_3_ and **c** Bi–Bi_2_O_2_CO_3_

**Fig. 2:**
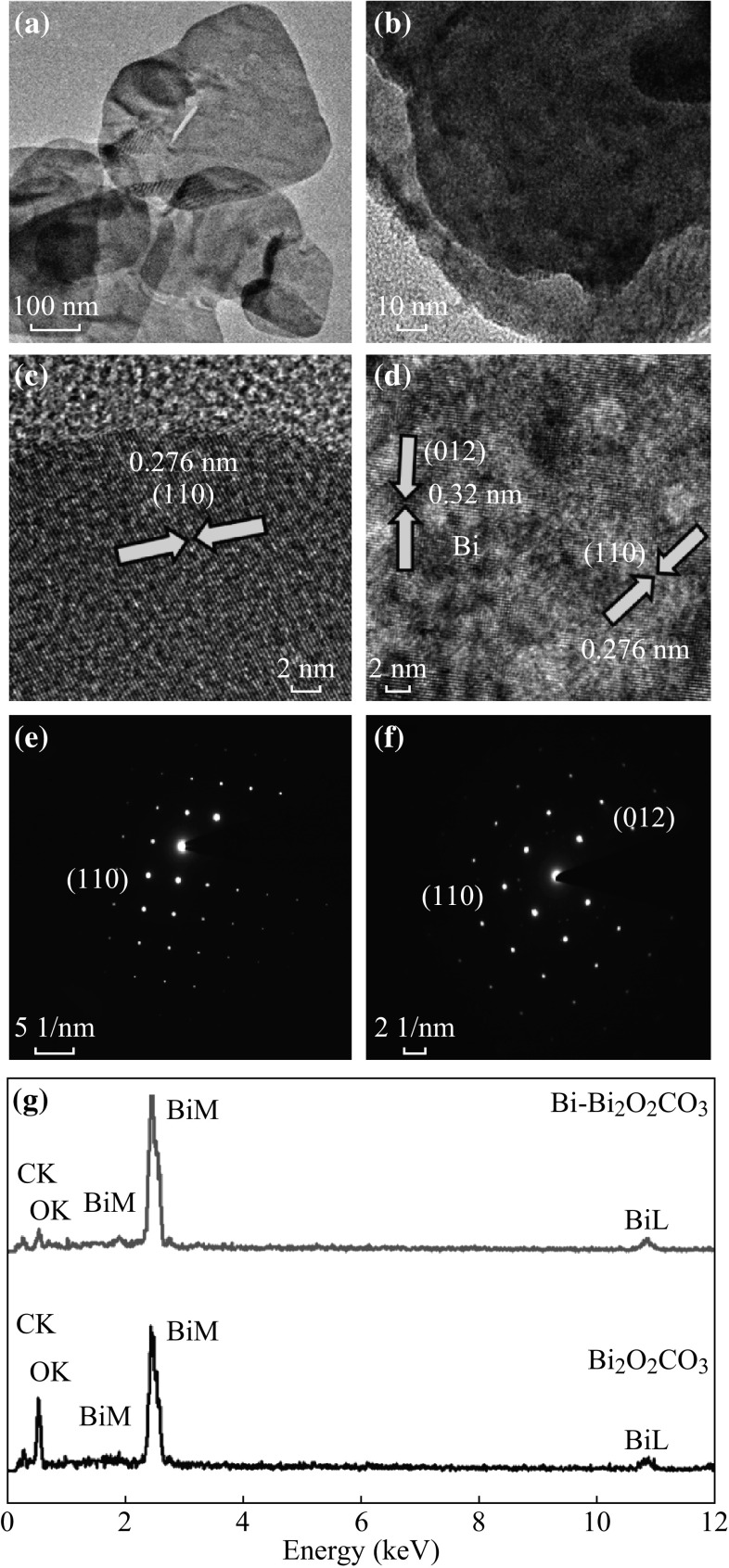
TEM images of **a** Bi_2_O_2_CO_3_ and **b** Bi–Bi_2_O_2_CO_3_. HRTEM images of **c** Bi_2_O_2_CO_3_ and **d** Bi–Bi_2_O_2_CO_3_. SAED patterns of **e** Bi_2_O_2_CO_3_ and **f** Bi–Bi_2_O_2_CO_3_. **g** EDAX spectrum of Bi_2_O_2_CO_3_ and Bi–Bi_2_O_2_CO_3_

Figure 1a shows XRD patterns of the as-synthesized Bi_2_O_2_CO_3_ and Bi–Bi_2_O_2_CO_3_. The diffraction pattern of Bi_2_O_2_CO_3_ is perfectly indicated as a tetragonal Bi_2_O_2_CO_3_ phase. After the addition of ethanol, the XRD pattern of the Bi–Bi_2_O_2_CO_3_ sample is also indexed to the Bi_2_O_2_CO_3_ phase (JCPDS card no. 41-1488) [44]. No characteristic peak for Bi nanoparticles in Bi–Bi_2_O_2_CO_3_ was observed, probably due to low content of Bi. Similar results were reported in previously literatures based on metal/semiconductor photocatalyst [35, 45, 46]. As shown in Fig. 1b, c, the SEM images of Bi_2_O_2_CO_3_ and Bi-Bi_2_O_2_CO_3_ reveal a large sheet-like morphology with a width from 50 to 600 nm. After decoration of Bi on the Bi_2_O_2_CO_3_ nanosheets, no significant structural and morphological change was observed. The smooth sheet-like morphology of Bi–Bi_2_O_2_CO_3_ indicates a uniform distribution of Bi nanoparticles on the surface of Bi_2_O_2_CO_3_. Morphology and crystallinity of Bi_2_O_2_CO_3_ and Bi–Bi_2_O_2_CO_3_ were further examined via TEM and HRTEM as shown in Fig. 2a–d. The TEM image of Bi–Bi_2_O_2_CO_3_ shows a uniform distribution of Bi nanoparticles on the surface of the Bi_2_O_2_CO_3_ nanosheets. The HRTEM images of Bi_2_O_2_CO_3_ and Bi–Bi_2_O_2_CO_3_ exhibit a high crystallinity of Bi_2_O_2_CO_3_ nanosheet and Bi nanoparticles as given in Fig. 2c, d. The inter-planar distance between the fringes are about 0.276 and 0.32 nm, which correspond to the (110) crystal plane of Bi_2_O_2_CO_3_ and (012) crystal plane of Bi nanoparticles, respectively [38, 47]. The selected area electron diffraction (SAED) pattern obtained from the HRTEM images (Fig. 2e, f) demonstrates further the well-crystallinity. From the EDAX measurement shown in Fig. 2g, the at.% ratio of Bi and O is 1:5 for Bi_2_O_2_CO_3_ whereas 2:3 for Bi–Bi_2_O_2_CO_3_. The XPS studies of Bi_2_O_2_CO_3_ and Bi–Bi_2_O_2_CO_3_ were well documented in earlier studies [18, 27, 31, 48]. In those studies, they concluded that the O 1s peak centered at 530.5 eV ascribed to Bi–O bonds in Bi_2_O_2_CO_3_, while peaks at 284.8 and 288.7 eV were the characteristic peaks of adventitious carbon species and CO_3_^2−^ in Bi_2_O_2_CO_3_. Peaks around 157.0 and 162.3 eV were assigned to the formation of Bi metal present in the heterostructure. The other characterizations on Bi_2_O_2_CO_3_ and Bi–Bi_2_O_2_CO_3_ related materials [18, 27, 31, 48] including HRTEM and EDAX are consistent with our experimental observations.

**Fig. 3:**
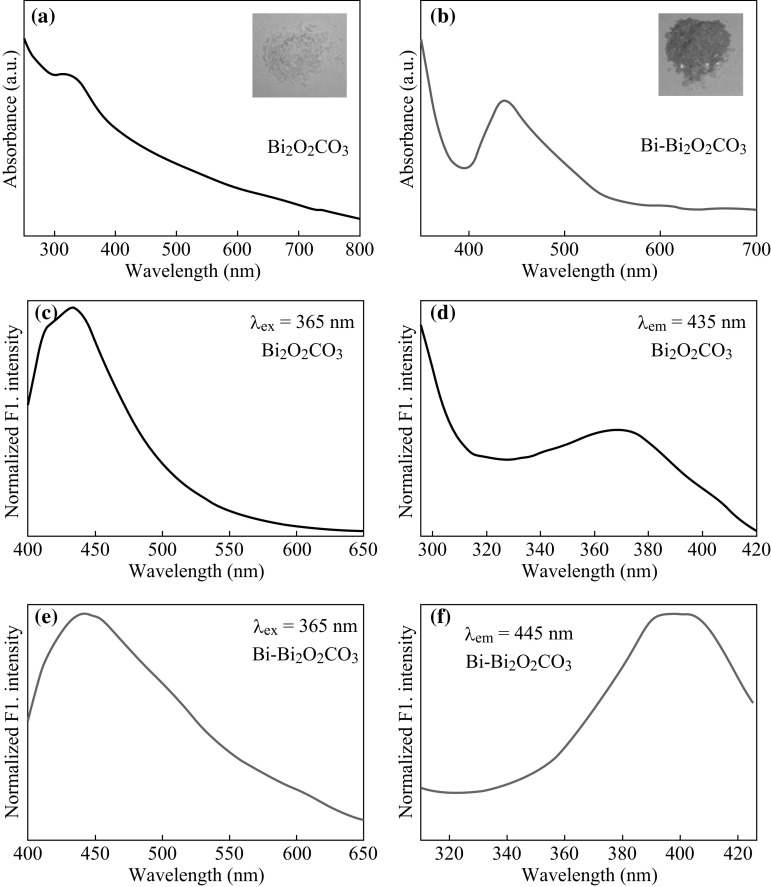
UV-Vis absorption spectrum of **a** Bi_2_O_2_CO_3_ and **b** Bi–Bi_2_O_2_CO_3_ (*Inset* shows the image of Bi_2_O_2_CO_3_ and Bi–Bi_2_O_2_CO_3_). Normalized steady-steady PL spectrum of **c** Bi_2_O_2_CO_3_ and **e** Bi–Bi_2_O_2_CO_3_. The excitation spectrum of Bi_2_O_2_CO_3_ **d** and Bi–Bi_2_O_2_CO_3_ **f** at different PL maxima

**Fig. 4:**
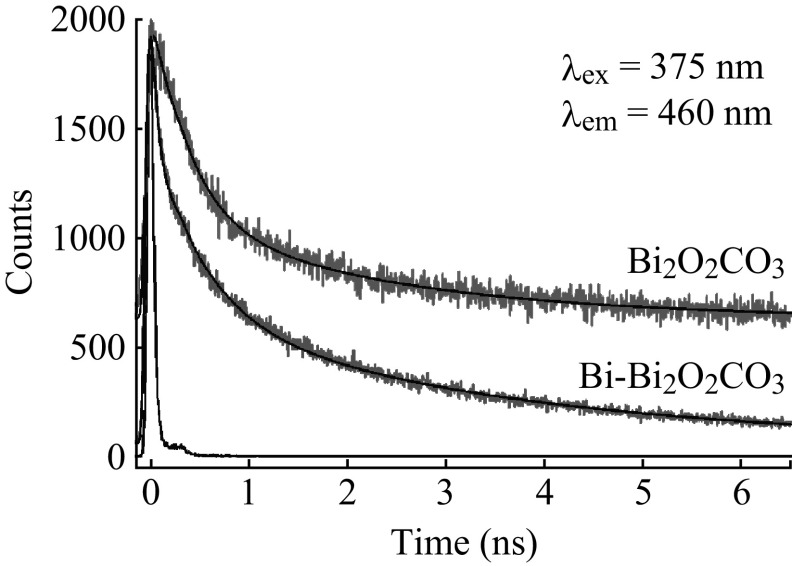
Picosecond-resolved PL transients of Bi_2_O_2_CO_3_ and Bi–Bi_2_O_2_CO_3_ measured at *λ*_em_ = 460 nm upon *λ*_ex_ = 375 nm

Figure 3a shows UV–Vis absorption spectra of Bi_2_O_2_CO_3_ and Bi–Bi_2_O_2_CO_3_. The Bi_2_O_2_CO_3_ shows absorption peak at 360 nm and long tail over 800 nm due to scattering of the nanoparticles presented in the solution, which consists with earlier reports [49, 50]. After formation of heterojunction Bi–Bi_2_O_2_CO_3_, an enhancement of absorption in the visible region was observed due to the presence of Bi nanoparticles, and surface plasmon absorption around 500 nm was found as shown in Fig. 3b. The SPR of non-noble metal Bi in the near ultraviolet and visible region were reported by different groups [51, 52]. Notably, such absorption enhancement in the visible region is also according to the color change of the samples as shown in the inset of Fig. 3a, b. Thus, formation of Bi nanoparticles on the surface of Bi_2_O_2_CO_3_ nanosheets results in an enhancement of absorption over the entire UV–Vis region. The photoluminescences of Bi_2_O_2_CO_3_ and Bi–Bi_2_O_2_CO_3_ exhibit emission around 400–550 nm upon excitation at 365 nm as shown in Fig. 3c, e. Picosecond-resolved fluorescence was studied to investigate the detailed photophysical properties of the heterostructure after decoration of Bi nanoparticles on the Bi_2_O_2_CO_3_ nanosheets. The fluorescence decay of Bi_2_O_2_CO_3_ and Bi–Bi_2_O_2_CO_3_ was determined at 460 nm upon excitation by 375 nm  laser source (Fig. 4) and tabulated in Table 1.

**Table 1:** Lifetimes of picosecond time-resolved PL transients of Bi_2_O_2_CO_3_ and Bi–Bi_2_O_2_CO_3_ detected at 460 nm PL maxima uopn excitation at 375 nm wavelength

System	*τ*_1_ (ps)	*τ*_2_ (ps)	*τ*_3_ (ps)	*τ*_avg_ (ns)
Bi_2_O_2_CO_3_	343 (71%)	3500 (29%)		1.25
Bi–Bi_2_O_2_CO_3_	50 (58%)	394 (25%)	3400 (17%)	0.70

The values in parentheses represent the relative weight percentages of the time components

The fluorescence decay of Bi_2_O_2_CO_3_ shows two components of 343 ps and 3.5 ns along with an average lifetime of 1.25 ns. After decoration of Bi nanoparticles, the average time of Bi–Bi_2_O_2_CO_3_ decreases to 0.70 ns. Thus, the faster component of 50 ps is attributed to the excited state electron transfer from Bi_2_O_2_CO_3_ to Bi. The obvious decrease in fluorescence lifetime of the heterostructures suggests that the decoration of Bi nanoparticles on the Bi_2_O_2_CO_3_ nanosheets can act as electron sink and therefore contribute to electron–hole separation. Such kind of metal-semiconductor heterojunctions facilitates in a remarkable way of the decline in the recombination of electron–hole pairs and is useful to enhance solar energy utilization [53–55].

**Fig. 5:**
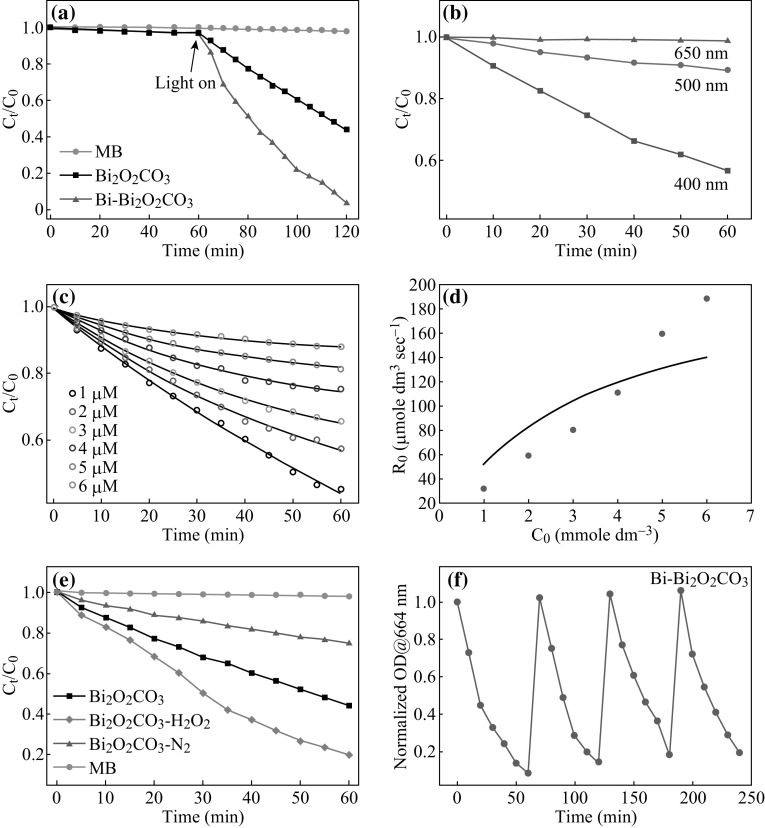
**a** Photocatalytic degradation of MB under visible light illumination. **b** Photocatalytic degradation of MB by Bi_2_O_2_CO_3_ at different wavelength. **c** C_t_/C_0_ versus time with various concentrations of methylene blue by Bi_2_O_2_CO_3_. **d** Langmuir–Hinshelwood plot (L–H) for photocatalytic degradation of methylene blue using Bi_2_O_2_CO_3_ (*solid line* is the model fitting and *solid circles* are experimental data). **e** Photodegradation of MB over Bi_2_O_2_CO_3_ and Bi–Bi_2_O_2_CO_3_ under conventional condition, presence of H_2_O_2_ and N_2_ into the solution. **f** A recyclability study of Bi–Bi_2_O_2_CO_3_ under visible light illumination

The photocatalytic activities of Bi_2_O_2_CO_3_ and Bi–Bi_2_O_2_CO_3_ were evaluated by photodegradation of the model organic contaminant MB under visible light illumination. However, in our case the as-prepared Bi_2_O_2_CO_3_ and Bi–Bi_2_O_2_CO_3_ have insignificant photocatalytic activity in dark (Fig. 5a). During the photocatalytic reaction, MB forms a well-known colorless product leucomethylene blue (LMB) [56, 57] as expressed in Eq. 2.

2 $$2{\text{MB}}\; + \;2{\text{e}}^{ - } \; + {\text{H}}^{ + } \; = \;{\text{MB}}\; + \;{\text{LMB}}$$

**Fig. 6:**
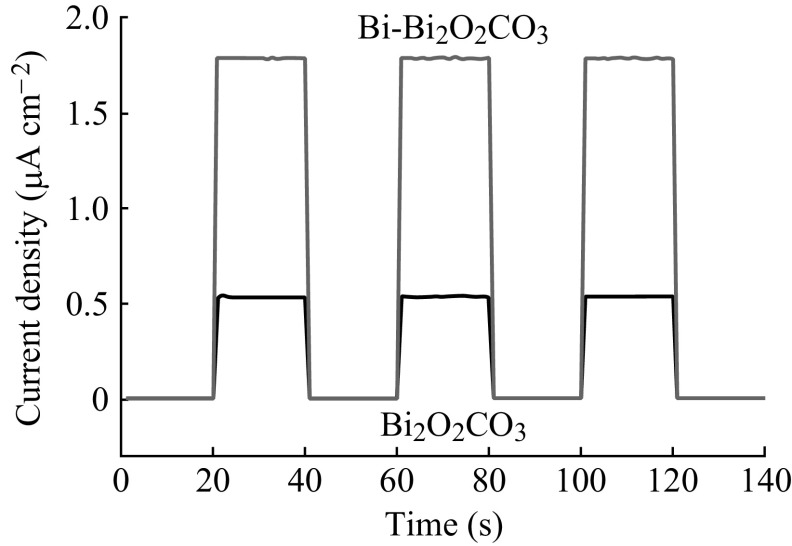
Current–time curves of electrodes made of pure Bi_2_O_2_CO_3_ and Bi–Bi_2_O_2_CO_3_ heterojunction

Figure 5a shows changes in MB concentration as a function of time in presence and absence of photocatalysts. With our experimental time window, MB has less than 10% degradation under light illumination in the absence of photocatalysts. In contrast, Bi_2_O_2_CO_3_ nanosheets show an enhanced photocatalytic activity and 60% of MB was degraded after 60 min illumination. One can see that presence of Bi nanoparticles on the Bi_2_O_2_CO_3_ nanosheets further enhance photocatalytic activity (100%) compared to Bi_2_O_2_CO_3_ nanosheets (60%). Figure 5b shows photocatalysis of methylene blue (MB) at different wavelength by Bi_2_O_2_CO_3_. Insignificant photocatalysis at 650 nm (MB absorbance maxima 660 nm) indicates that MB is unable to photosensitize Bi_2_O_2_CO_3_. Thus photocatalysis predominately takes place via sensitization of Bi_2_O_2_CO_3_. In order to find out the effect of the surface on photocatalysis, the Langmuir–Hinshelwood (L–H) kinetics was studied using different concentrations of MB (Fig. 5c). As shown in Fig. 5d, a significant deviation of the model (solid line) from experimental data is evident. The observation indicates that surface adsorption of the model pollutant plays insignificant role in the photodegradation. In order to investigate the catalytic pathway, we further studied the photocatalytic activity of Bi_2_O_2_CO_3_ in the presence of a radical initiator (H_2_O_2_) and radical quencher (N_2_ bubbling) separately (Fig. 5e). In fact, in the presence of H_2_O_2_ under solar light illumination increases generation of OH**·** which eventually increases the photocatalytic activity of Bi_2_O_2_CO_3_ for degradation of MB. This demonstrates the role of reactive oxygen species (ROS) in the degradation of MB [58]. The photodegradation efficiency of Bi_2_O_2_CO_3_ decreases with N_2_ bubbling in the solution, so O_2_ primarily acts as an efficient electron trap, leading to the generation of O_2_^**−**^radicals during photocatalytic reaction [59]. From the application point of view, photochemical stability and durability of photocatalysts are significant during photocatalytic reaction [60]. To further test photocatalytic performance of the as-prepared heterojunction Bi–Bi_2_O_2_CO_3_ photocatalyst, recycling experiment was carried out under repeated irradiation. Figure 5f shows the repeated photocatalytic activity of Bi–Bi_2_O_2_CO_3_. The results indicate that the rate remains similar after four consecutive cycles, implying that the obtained Bi–Bi_2_O_2_CO_3_ heterojunction photocatalyst has high stability and no photocorrosion occurs during the photodegradation of MB. Photocurrent measurement was carried out under solar light illumination to investigate the efficient electron–hole separation. The photocurrent of Bi–Bi_2_O_2_CO_3_ heterostructures is much higher than that of Bi_2_O_2_CO_3_ (see Fig. 6). This implies that the heterojunction shows an improved separation of photogenerated electron–hole pairs and can greatly facilitate its photocatalytic activity.

**Scheme 1:**
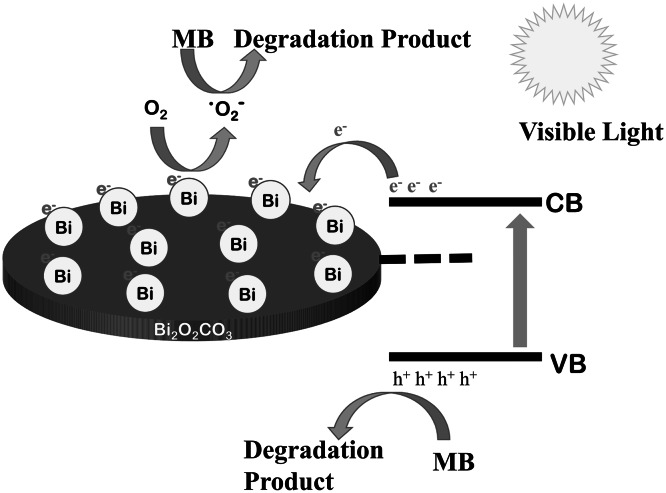
Schematic illustration of enhanced photocatalytic activity by Bi–Bi_2_O_2_CO_3_ heterojunction under visible light illumination

There are several reports which indicate that the enhancement in photocatalytic performance can be ascribed to the synergetic effects of many factors, such as hierarchical structure, surface area, interfacial charge transfer, and efficient separation of photoinduced electrons and holes [61–65]. In the present study, the enhanced photocatalytic performance for the Bi–Bi_2_O_2_CO_3_ photocatalyst can be ascribed to the formation of heterojunction between Bi nanoparticles and the surface of Bi_2_O_2_CO_3_ nanosheets. Furthermore, the Fermi level of Bi nanoparticles which acts as electron acceptors can be estimated to be about −0.17 eV as calculated by the work function of metallic bismuth of 4.22 eV [66–68]. Since the Fermi level of metallic Bi (−0.17 eV) is lower than the conduction band of Bi_2_O_2_CO_3_ (−1.40 eV) [27], the photogenerated electrons would probably transfer from Bi_2_O_2_CO_3_ to the deposited Bi nanoparticles and therefore promote the separation of photo-generated electrons and holes, effectively. After the separation of electrons and holes, these two kinds of photogenerated charge carriers would be transformed into reactive species that are responsible for promoting photocatalytic activity. Based on the above investigations, a schematic illustration is proposed as shown in Scheme 1.

## Conclusion

We successfully synthesized Bi–Bi_2_O_2_CO_3_ heterojunction by a one-step hydrothermal method. Detailed spectroscopic investigations reveal that ultrafast photo-induced charge separation in the Bi–Bi_2_O_2_CO_3_ heterojunction is conducive for enhanced solar energy conversion. As a potential prototype application, we found enhanced photocatalytic activity of the heterostructure using MB as a model organic contaminant under visible light illumination. The efficient separation of photoinduced electron–hole pairs in the heterojunction was further proved by photocurrent measurement. Moreover, this work not only provides cost effective procedure to prepare efficient photocatalyst with high stability but also opens up a new field for bismuth containing heterostructures with several future applications.

## References

[1] Chong MN, Jin B, Chow CWK, Saint C (2010). Recent developments in photocatalytic water treatment technology: a review. Water Res..

[2] Hoffmann MR, Martin ST, Choi W, Bahnemann DW (1995). Environmental applications of semiconductor photocatalysis. Chem. Rev..

[3] Dong S, Feng J, Fan M, Pi Y, Hu L, Han X, Liu M, Sun J (2015). Recent developments in heterogeneous photocatalytic water treatment using visible light-responsive photocatalysts: a review. RSC Adv..

[4] Lonkar Sunil P, Pillai Vishnu V, Stephen Samuel, Abdala Ahmed, Mittal Vikas (2016). Facile in situ fabrication of nanostructured graphene-CuO hybrid with hydrogen sulfide removal capacity. Nano-Micro Lett..

[5] Kar P, Sardar S, Ghosh S, Parida MR, Liu B, Mohammed OF, Lemmens P, Pal SK (2015). Nano surface engineering of Mn_2_O_3_ for potential light-harvesting application. J. Mater. Chem. C.

[6] T.K. Maji, D. Bagchi, P. Kar, D. Karmakar, S.K. Pal, Enhanced charge separation through modulation of defect-state in wide band-gap semiconductor for potential photocatalysis application: Ultrafast spectroscopy and computational studies. J. Photochem. Photobiol. A **332**, 391–398 (2017). doi:10.1016/j.jphotochem.2016.09.017

[7] Wang Y, Wang Q, Zhan X, Wang F, Safdar M, He J (2013). Visible light driven type II heterostructures and their enhanced photocatalysis properties: a review. Nanoscale.

[8] Liqiang J, Xiaojun S, Jing S, Weimin C, Zili X, Yaoguo D, Honggang F (2003). Review of surface photovoltage spectra of nano-sized semiconductor and its applications in heterogeneous photocatalysis. Sol. Energy Mater. Sol. Cells.

[9] Huang H, Li X, Wang J, Dong F, Chu PK, Zhang T, Zhang Y (2015). Anionic group self-doping as a promising strategy: band-gap engineering and multi-functional applications of high-performance CO_32_-doped Bi_2_O_2_CO_3_. ACS Catal..

[10] Huang H, Han X, Li X, Wang S, Chu PK, Zhang Y (2015). Fabrication of multiple heterojunctions with tunable visible-light-active photocatalytic reactivity in BiOBr–BiOI full-range composites based on microstructure modulation and band structures. ACS Appl. Mater. Interfaces.

[11] Huang H, Xiao K, He Y, Zhang T, Dong F, Du X, Zhang Y (2016). In situ assembly of BiOI@Bi_12_O_17_Cl_2_ p-n junction: charge induced unique front-lateral surfaces coupling heterostructure with high exposure of BiOI {001} active facets for robust and nonselective photocatalysis. Appl. Catal. B.

[12] Huang H, Liu K, Chen K, Zhang Y, Zhang Y, Wang S (2014). Ce and F comodification on the crystal structure and enhanced photocatalytic activity of Bi_2_WO_6_ photocatalyst under visible light irradiation. J. Phys. Chem. C.

[13] He Q, Huang S, Wang C, Qiao Q, Liang N, Xu M, Chen W, Zai J, Qian X (2015). The role of Mott-Schottky heterojunctions in Ag–Ag8SnS6 as counter electrodes in dye-sensitized solar cells. Chem. Sus. Chem..

[14] Huang S, He Q, Zai J, Wang M, Li X, Li B, Qian X (2015). The role of Mott-Schottky heterojunctions in PtCo-Cu_2_ZnGeS_4_ as counter electrodes in dye-sensitized solar cells. Chem. Commun..

[15] Jin L, Zhu G, Hojamberdiev M, Luo X, Tan C, Peng J, Wei X, Li J, Liu P (2014). A plasmonic Ag–AgBr/Bi_2_O_2_CO_3_ composite photocatalyst with enhanced visible-light photocatalytic activity. Ind. Eng. Chem. Res..

[16] Zhu G, Hojamberdiev M, Katsumata K-I, Cai X, Matsushita N, Okada K, Liu P, Zhou J (2013). Heterostructured Fe_3_O_4_/Bi_2_O_2_CO_3_ photocatalyst: synthesis, characterization and application in recyclable photodegradation of organic dyes under visible light irradiation. Mater. Chem. Phys..

[17] Hameed A, Montini T, Gombac V, Fornasiero P (2008). Surface phases and photocatalytic activity correlation of Bi_2_O_3_/Bi_2_O_4-x_ nanocomposite. J. Am. Chem. Soc..

[18] Xiao S, Li Y, Hu J, Li H, Zhang X, Liu L, Lian J (2015). One-step synthesis of nanostructured Bi-Bi_2_O_2_CO_3_-ZnO composites with enhanced photocatalytic performance. Cryst. Eng. Comm..

[19] Cai P, Zhou S, Ma D, Liu S, Chen W, Huang S (2015). Fe_2_O_3_-modified porous BiVO_4_ nanoplates with enhanced photocatalytic activity. Nano-Micro Lett..

[20] Wang W, Wang J, Wang Z, Wei X, Liu L, Ren Q, Gao W, Liang Y, Shi H (2014). p-n junction CuO/BiVO_4_ heterogeneous nanostructures: synthesis and highly efficient visible-light photocatalytic performance. Dalton Trans..

[21] Zhang Y, Zhu G, Hojamberdiev M, Gao J, Hao J, Zhou J, Liu P (2016). Synergistic effect of oxygen vacancy and nitrogen doping on enhancing the photocatalytic activity of Bi_2_O_2_CO_3_ nanosheets with exposed {001} facets for the degradation of organic pollutants. Appl. Surf. Sci..

[22] Greaves C, Blower SK (1988). Structural relationships between Bi_2_O_2_CO_3_ and β-Bi_2_O_3_. Mater. Res. Bull..

[23] Madhusudan P, Zhang J, Cheng B, Liu G (2013). Photocatalytic degradation of organic dyes with hierarchical Bi_2_O_2_CO_3_ microstructures under visible-light. CrystEngComm.

[24] Zhao T, Zai J, Xu M, Zou Q, Su Y, Wang K, Qian X (2011). Hierarchical Bi_2_O_2_CO_3_ microspheres with improved visible-light-driven photocatalytic activity. CrystEngComm.

[25] Madhusudan P, Yu J, Wang W, Cheng B, Liu G (2012). Facile synthesis of novel hierarchical graphene-Bi_2_O_2_CO_3_ composites with enhanced photocatalytic performance under visible light. Dalton Trans..

[26] Xu Y-S, Zhang W-D (2013). Anion exchange strategy for construction of sesame-biscuit-like Bi_2_O_2_CO_3_/Bi_2_MoO_6_ nanocomposites with enhanced photocatalytic activity. Appl. Catal. B.

[27] Zhao Z, Zhou Y, Wang F, Zhang K, Yu S, Cao K (2015). Polyaniline-decorated 001 facets of Bi_2_O_2_CO_3_ nanosheets: in situ oxygen vacancy formation and enhanced visible light photocatalytic activity. ACS Appl. Mater. Interfaces.

[28] Liang N, Wang M, Jin L, Huang S, Chen W (2014). Highly efficient Ag_2_O/Bi_2_O_2_CO_3_ p-n heterojunction photocatalysts with improved visible-light responsive activity. ACS Appl. Mater. Interfaces.

[29] Peng S, Li L, Tan H, Wu Y, Cai R (2013). Monodispersed Ag nanoparticles loaded on the PVP-assisted synthetic Bi_2_O_2_CO_3_ microspheres with enhanced photocatalytic and supercapacitive performances. J. Mater. Chem. A.

[30] Wang R, Li X, Cui W, Zhang Y, Dong F (2015). In situ growth of Au nanoparticles on 3D Bi_2_O_2_CO_3_ for surface plasmon enhanced visible light photocatalysis. New J. Chem..

[31] Dong F, Li Q, Sun Y, Ho W-K (2014). Noble metal-like behavior of plasmonic Bi particles as a cocatalyst deposited on (BiO)_2_CO_3_ microspheres for efficient visible light photocatalysis. ACS Catal..

[32] Safardoust-Hojaghan H, Salavati-Niasari M, Motaghedifard MH, Hosseinpour-Mashkani SM (2015). Synthesis of micro sphere-like bismuth nanoparticles by microwave assisted polyol method; designing a novel electrochemical nanosensor for ultra-trace measurement of Pb^2+^ ions. New J. Chem..

[33] Wang Z, Jiang C, Huang R, Peng H, Tang X (2014). Investigation of optical and photocatalytic properties of bismuth nanospheres prepared by a facile thermolysis method. J. Phys. Chem. C.

[34] Dong F, Xiong T, Sun Y, Zhao Z, Zhou Y, Feng X, Wu Z (2014). A semimetal bismuth element as a direct plasmonic photocatalyst. Chem. Commun..

[35] Chen D, Zhang M, Lu Q, Chen J, Liu B, Wang Z (2015). Facile synthesis of Bi/BiOCl composite with selective photocatalytic properties. J. Alloys Compd..

[36] Huang Y, Wang W, Zhang Q, Cao J-J, Huang R-J, Ho W, Lee SC (2016). In situ fabrication of α-Bi_2_O_3_/(BiO)_2_CO_3_ nanoplate heterojunctions with tunable optical property and photocatalytic activity. Sci. Rep..

[37] Chang C, Zhu L, Fu Y, Chu X (2013). Highly active Bi/BiOI composite synthesized by one-step reaction and its capacity to degrade bisphenol A under simulated solar light irradiation. Chem. Eng. J..

[38] Gao M, Zhang D, Pu X, Li H, Lv D, Zhang B, Shao X (2015). Facile hydrothermal synthesis of Bi/BiOBr composites with enhanced visible-light photocatalytic activities for the degradation of rhodamine B. Sep. Purif. Technol..

[39] Chen Y, Chen D, Chen J, Lu Q, Zhang M, Liu B, Wang Q, Wang Z (2015). Facile synthesis of Bi nanoparticle modified TiO_2_ with enhanced visible light photocatalytic activity. J. Alloys Compd..

[40] Liu X, Cao H, Yin J (2011). Generation and photocatalytic activities of Bi@Bi_2_O_3_ microspheres. Nano Res..

[41] Sardar S, Kar P, Sarkar S, Lemmens P, Pal SK (2015). Interfacial carrier dynamics in PbS-ZnO light harvesting assemblies and their potential implication in photovoltaic/photocatalysis application. Sol. Energ. Mat. Sol. Cells.

[42] Sarkar PK, Polley N, Chakrabarti S, Lemmens P, Pal SK (2016). Nanosurface energy transfer based highly selective and ultrasensitive “Turn on” fluorescence mercury sensor. ACS Sens..

[43] Sardar S, Kar P, Remita H, Liu B, Lemmens P, Kumar Pal S, Ghosh S (2015). Enhanced charge separation and FRET at heterojunctions between semiconductor nanoparticles and conducting polymer nanofibers for efficient solar light harvesting. Sci. Rep..

[44] Sun Y, Zhao Z, Dong F, Zhang W (2015). Mechanism of visible light photocatalytic NO_x_ oxidation with plasmonic Bi cocatalyst-enhanced (BiO)_2_CO_3_ hierarchical microspheres. Phys. Chem. Chem. Phys..

[45] Chen JLT, Nalla V, Kannaiyan G, Mamidala V, Ji W, Vittal JJ (2014). Synthesis and nonlinear optical switching of Bi_2_S_3_ nanorods and enhancement in the NLO response of Bi_2_S_3_@Au nanorod-composites. New J. Chem..

[46] Lim SP, Pandikumar A, Huang NM, Lim HN (2015). Facile synthesis of Au@TiO_2_ nanocomposite and its application as a photoanode in dye-sensitized solar cells. RSC Adv..

[47] Cheng G, Yang H, Rong K, Lu Z, Yu X, Chen R (2010). Shape-controlled solvothermal synthesis of bismuth subcarbonate nanomaterials. J. Solid State Chem..

[48] Zhu G, Liu Y, Hojamberdiev M, Han J, Rodriguez J, Bilmes SA, Liu P (2015). Thermodecomposition synthesis of porous β-Bi_2_O_3_/Bi_2_O_2_CO_3_ heterostructured photocatalysts with improved visible light photocatalytic activity. New J. Chem..

[49] Lin S, Liu L, Liang Y, Cui W, Zhang Z (2016). Oil-in-water self-assembled synthesis of Ag@AgCl nano-particles on flower-like Bi_2_O_2_CO_3_ with enhanced visible-light-driven photocatalytic activity. Materials.

[50] Siegel J, Kvítek O, Ulbrich P, Kolská Z, Slepička P, Švorčík V (2012). Progressive approach for metal nanoparticle synthesis. Mater. Lett..

[51] Weng S, Chen B, Xie L, Zheng Z, Liu P (2013). Facile in situ synthesis of a Bi/BiOCl nanocomposite with high photocatalytic activity. J. Mater. Chem. A.

[52] Toudert J, Serna R, Jiménez de Castro M (2012). Exploring the optical potential of nano-bismuth: tunable surface plasmon resonances in the near ultraviolet-to-near infrared range. J. Phys. Chem. C.

[53] Cushing SK, Wu N (2016). Progress and perspectives of plasmon-enhanced solar energy conversion. J. Phys. Chem. Lett..

[54] Li J, Cushing SK, Meng F, Senty TR, Bristow AD, Wu N (2015). Plasmon-induced resonance energy transfer for solar energy conversion. Nat. Photon..

[55] Costi R, Saunders AE, Elmalem E, Salant A, Banin U (2008). Visible light-induced charge retention and photocatalysis with hybrid CdSe–Au nanodumbbells. Nano Lett..

[56] Yogi C, Kojima K, Wada N, Tokumoto H, Takai T, Mizoguchi T, Tamiaki H (2008). Photocatalytic degradation of methylene blue by TiO_2_ film and Au particles-TiO_2_ composite film. Thin Solid Films.

[57] Mills A, Wang J (1999). Photobleaching of methylene blue sensitised by TiO_2_: an ambiguous system?. J. Photochem. Photobiol. A.

[58] Giri A, Goswami N, Pal M, Zar Myint MT, Al-Harthi S, Singha A, Ghosh B, Dutta J, Pal SK (2013). Rational surface modification of Mn_3_O_4_ nanoparticles to induce multiple photoluminescence and room temperature ferromagnetism. J. Mater. Chem. C.

[59] Park Y, Lee S-H, Kang SO, Choi W (2010). Organic dye-sensitized TiO_2_ for the redox conversion of water pollutants under visible light. Chem. Commun..

[60] Ai Z, Ho W, Lee S, Zhang L (2009). Efficient photocatalytic removal of NO in indoor air with hierarchical bismuth oxybromide nanoplate microspheres under visible light. Environ. Sci. Technol..

[61] Dong F, Sun Y, Fu M, Wu Z, Lee SC (2012). Room temperature synthesis and highly enhanced visible light photocatalytic activity of porous BiOI/BiOCl composites nanoplates microflowers. J. Hazard. Mater..

[62] Xiang Q, Yu J, Jaroniec M (2012). Graphene-based semiconductor photocatalysts. Chem. Soc. Rev..

[63] Li Q, Liu H, Dong F, Fu M (2013). Hydrothermal formation of N-doped (BiO)_2_CO_3_ honeycomb-like microspheres photocatalysts with bismuth citrate and dicyandiamide as precursors. J. Colloid Interface Sci..

[64] Yu S, Huang H, Dong F, Li M, Tian N, Zhang T, Zhang Y (2015). Synchronously achieving plasmonic bi metal deposition and I^– ^doping by utilizing BiOIO_3_ as the self-sacrificing template for high-performance multifunctional applications. ACS Appl. Mater. Interfaces.

[65] Guo Y, Zhang Y, Tian N, Huang H (2016). Homogeneous {001}-BiOBr/Bi heterojunctions: facile controllable synthesis and morphology-dependent photocatalytic activity. ACS Sustain. Chem. Eng..

[66] Subramanian V, Wolf EE, Kamat PV (2004). Catalysis with TiO_2_/gold nanocomposites. Effect of metal particle size on the fermi level equilibration. J. Am. Chem. Soc..

[67] Trasatti S (1972). Electronegativity, work function, and heat of adsorption of hydrogen on metals. J. Chem. Soc. Faraday Trans. I.

[68] Yu Y, Cao C, Liu H, Li P, Wei F, Jiang Y, Song W (2014). A Bi/BiOCl heterojunction photocatalyst with enhanced electron-hole separation and excellent visible light photodegrading activity. J. Mater. Chem. A.

